# Marker effects and heritability estimates using additive-dominance genomic architectures via artificial neural networks in *Coffea canephora*

**DOI:** 10.1371/journal.pone.0262055

**Published:** 2022-01-26

**Authors:** Ithalo Coelho de Sousa, Moysés Nascimento, Isabela de Castro Sant’anna, Eveline Teixeira Caixeta, Camila Ferreira Azevedo, Cosme Damião Cruz, Felipe Lopes da Silva, Emilly Ruas Alkimim, Ana Carolina Campana Nascimento, Nick Vergara Lopes Serão

**Affiliations:** 1 Department of Animal Science, Iowa State University, Ames, Iowa, United States of America; 2 Department of Statistics, Federal University of Viçosa, Viçosa, Minas Gerais, Brazil; 3 Rubber Tree and Agroforestry Systems Research Center, Campinas Agronomy Institute (IAC), Votuporanga, São Paulo, Brazil; 4 Brazilian Agricultural Research Corporation, Embrapa Coffee, Brasília, DF, Brazil; 5 Department of General Biology, Federal University of Viçosa, Viçosa, Minas Gerais, Brazil; 6 Department of Plant Science, Federal University of Viçosa, Viçosa, Minas Gerais, Brazil; 7 Federal University of Triangulo Mineiro, Iturama, Minas Gerais, Brazil; Government College University Faisalabad, PAKISTAN

## Abstract

Many methodologies are used to predict the genetic merit in animals and plants, but some of them require priori assumptions that may increase the complexity of the model. Artificial neural network (ANN) has advantage to not require priori assumptions about the relationships between inputs and the output allowing great flexibility to handle different types of complex non-additive effects, such as dominance and epistasis. Despite this advantage, the biological interpretability of ANNs is still limited. The aim of this research was to estimate the heritability and markers effects for two traits in *Coffea canephora* using an additive-dominance architecture ANN and to compare it with genomic best linear unbiased prediction (GBLUP). The data used consists of 51 clones of *C*. *canephora* varietal Conilon, 32 of varietal group Robusta and 82 intervarietal hybrids. From this, 165 phenotyped individuals were genotyped for 14,387 SNPs. Due to the high computational cost of ANNs, we used Bagging decision tree to reduce the dimensionality of the data, selecting the markers that accumulated 70% of the total importance. An ANN with three hidden layers was run, each varying from 1 to 40 neurons summing 64,000 neural networks. The network architectures with the best predictive ability were selected. The best architectures were composed by 4, 15, and 33 neurons in the first, second and third hidden layers, respectively, for yield, and by 13, 20, and 24 neurons, respectively for rust resistance. The predictive ability was greater when using ANN with three hidden layers than using one hidden layer and GBLUP, with 0.72 and 0.88 for yield and coffee leaf rust resistance, respectively. The concordance rate (CR) of the 10% larger markers effects among the methods varied between 10% and 13.8%, for additive effects and between 5.4% and 11.9% for dominance effects. The narrow-sense ($h_{a}^{2}$) and dominance-only ($h_{d}^{2}$) heritability estimates were 0.25 and 0.06, respectively, for yield, and 0.67 and 0.03, respectively for rust resistance. The ANN was able to estimate the heritabilities from an additive-dominance genomic architectures and the ANN with three hidden layers obtained best predictive ability when compared with those obtained from GBLUP and ANN with one hidden layer.

## Introduction

The interest in semi- and non-parametric statistical methods for genome-enabled prediction is increasing [1]. Methodologies based on machine learning, as Artificial Neural Networks (ANN), has been successfully used to predict the genetic merit in animals [2, 3] and plants [4, 5]. ANN is a methodology inspired by the biological behavior of human brain. ANN comprises layers divided into units called neurons. Each neuron’s output is expressed as the sum of inputs to a neuron, regulating specific weights for the predictor variables through linear and nonlinear activation functions [1, 6]. ANN have been applied for genomic prediction of complex traits in some crops as maize, eucalypt [7], soybean [8] and wheat [9]. This approach does not require making a priori assumptions about the relationships between inputs (SNP markers) and the output (phenotypic observations). The non-priori assumptions allow for great flexibility to handle different types of complex non-additive effects, such as dominance and epistasis [1, 10, 11].

Despite this advantage, reports about the biological interpretation from the marker effects and genetic parameter (i.e., heritability) estimates are limited to the best of our knowledge. Glória et al, [1] using simulated data, aimed to evaluate Bayesian regularized ANNs’ predictive performance and exploit SNP effects and heritability estimates. Considering only additive effects, the authors observed that based on the predictive ability and estimates of the heritabilities, the best ANN presented similar results to those obtained by Ridge Regression BLUP (RR_BLUP) and Bayesian Lasso (BLASSO).

For some species, for example, maize, eucalyptus, cotton, rice, pinus, and coffee [12–17], where there is commercial interest in hybrids and heterosis, the contribution of dominance presents high importance [16]. Coffee is globally one of the most important export crops and is a part of the economy in more than 50 countries in Latin America, Africa, and Asia. Besides the yield, traits associated with resistance to coffee rust are important in the selection in coffee, since the coffee production can be reduced in the presence of this disease [18]. Therefore, the identification of cultivars having resistance for diseases can improve the productivity of the culture. Despite its relevance, the effective selection of new cultivars depends on the ability to consider genomic models, which correctly represent complex traits with additive and dominance effects. Therefore, methods considering dominance effects, different numbers of layers, and neurons to exploit SNP effects and heritability can bring new insights for genomic selection in coffee.

Against this background, we aimed to exploit SNP effects and heritability from additive-dominance genomic model by ANN of traits associated with the yield and coffee leaf rust resistance, in *Coffea cenephora*. In addition, we predicted the individual genetic merits of the traits (yield and coffee leaf rust resistance) using ANN, and compared the predictive ability obtained for ANN and GBLUP for predicting genetic merit.

## Material and methods

### Phenotypic data

The used population consisted of 51 clones of *C*. *canephora* varietal group Conilon, 32 varietal group Robusta and 82 intervarietal hybrids. These hybrids were originated from crosses between five Conilon genotypes (males) and five Robusta (females), obtained in a partial diallel model [19]. The Conilon genetic material was obtained from the Capixaba Institute for Research, Technical Assistance, and Rural Extension (INCAPER, Vitória, ES, Brazil). The Robusta material was obtained from the Tropical Agronomic Research and Teaching Center (CATIE, Cartago, Turrialba, Costa Rica). This population composes the breeding program of the Agricultural Research Company of Minas Gerais (Epamig, Belo Horizonte, MG, Brazil) in partnership with the Federal University of Viçosa (UFV, Viçosa, Minas Gerais, Brazil) and the Brazilian Agricultural Research Company—Café (Embrapa Café, Oratório, Minas Gerais, Brazil).

Individuals were phenotyped for two traits, coffee leaf rust resistance and yield, for three years (2014 to 2016). Coffee leaf rust resistance (Hemileia vastatrix) was evaluated using a 5-point scale (1 = fully resistant, 5 = highly susceptible). The yield per coffee plant was evaluated by harvesting all fruits present in a genotype and measuring the total volume of freshly harvested coffee liters.

### SNP genotyping

DNA samples of 165 young and fully expanded leaves coffee were genotyped using the methodology described by Diniz et al. [20]. The concentration of DNA was verified in NanoDrop 2000, and its quality was evaluated in 1% agarose gel. The sample’s DNA concentration was standardized and sent to Rapid Genomics (Florida, Orlando, USA) for identification of SNP molecular markers. The data was genotyped using the Capture Seq methodologie [21], totalizing 14,387 markers.

Marker genotypes were coded according to the effects assumed. For additive effects, homozygous markers containing only alleles with minor frequency, the value is 0. For heterozygous markers, the value is 1, and for homozygous markers containing only alleles with major frequency, the value is 2. For dominant codification, we used 0 for homozygous marker and 1 for heterozygous marker.

### Phenotypic data analysis

Prior to genomic analyses, the phenotypic data of both traits were independently adjusted for systematic effects using Selegen REML/BLUP software [22] according to the following statistical model:

y=Xu+Tc+Wf+Zm+Qs+Sb+e (1)

where *y* is the observed phenotype; **μ** is the effect of the overall mean in each evaluation year (assumed as fixed effect) added to the general mean; **c** is the dominance effect of combination between the parents Conilon and Robusta (assumed as random effect and distributed as $N\sim I\sigma_{c}^{2}$); **f** is the additive effect of combination of the parent Robusta (assumed as random effect and distributed as $N\sim A\sigma_{f}^{2}$); ***m*** is the additive effect of combination of the parent Conilon (assumed as random effect and distributed as $N\sim A\sigma_{m}^{2}$); ***s*** is the effect of permanent environment of individuals (assumed as random effect and distributed as $N\sim I\sigma_{s}^{2}$); ***b*** is the effect of permanent environment of blocks (assumed as random effect and distributed as $N\sim I\sigma_{b}^{2}$); ***e*** is the residuals (assumed as random effect and distributed as $N\sim I\sigma_{e}^{2}$); and X, T, W, Z, Q, and S are the design matrices for the effects of *μ*, *c*, *f*, *m*, *s*, and, *b*, respectivaly. From this, adjusted phenotypes (Y*) were calculated as the sum of the estimates of random effects ***c***, ***f***, and ***m***, and the residual, and used for subsequent genomic analyses that were carried out in R [23].

### Genomic analyses

#### Genomic BLUP (GBLUP)

The additive dominance model for the REML/GBLUP (restricted maximum likelihood/genomic linear unbiased predictor) method is given by:

$$Y^{*}=Xb+Z\mu_{a}+Z\mu_{d}+e,$$ (2)

where Y* is the vector of adjusted phenotypic observations obtained in Eq (1), **b** is the vector of fixed effects, ***μ***_***a***_ is the vector of random of additive marker effects, ***μ***_***d***_ is the vector of random of dominance marker effects, **e** refers to the vector of random errors; and X, Z, are the design matrix. The variance structure is given by:

$$\left[\begin{array}{c} \mu_{a}\\ \mu_{d}\\ e \end{array}\right]\sim N\left(\left[\begin{array}{c} 0\\ 0\\ 0 \end{array}\right],\left[\begin{array}{ccc} G_{a}\sigma_{\mu_{a}}^{2} & 0 & 0\\ 0 & G_{d}\sigma_{\mu_{d}}^{2} & 0\\ 0 & 0 & I\sigma_{e}^{2} \end{array}\right]\right)$$

where ***G***_***a***_ and ***G***_***d***_ are the genomic relationship matrices for the additive and dominance effects, respectively, and ***I*** is the identity matrix.

An equivalent model [24] at the marker level is given by

$$Y^{*}=Xb+ZUm_{a}+ZSm_{d}+e,$$ (3)

where: ***μ***_***a***_ = **Um**_**a**_; Var(**Um**_**a**_) = **UI**$\sigma_{{\text{m}}_{\text{a}}}^{2}$**U’** = **UU**’$\sigma_{{\text{m}}_{\text{a}}}^{2}$;***μ***_***d***_ = ***Sm***_***d***_; Var(**S*m***_***d***_) = **SI**$\sigma_{m_{d}}^{2}$**S**’ = **SS**’$\sigma_{{\text{m}}_{\text{a}}}^{2}$; **X** is the design matrix for the vector **b** and **Z** is the design matrix for the vectors additive (**m**_**a**_) and dominance (**m**_**d**_) marker genetic effects. The variance components associated to these effects are $\sigma_{{\text{m}}_{\text{a}}}^{2}$ and $\sigma_{m_{d}}^{2}$, respectively. The quantity **m**_**a**_ in one locus is the allele substitution effect and is given by *m*_*a*_ = *α*_*i*_ = *a*_*i*_ + (*q*_*i*_–*p*_*i*_)*d*_*i*_, where *p*_*i*_ and *q*_*i*_ are allelic frequencies and *a*_*i*_ and *d*_*i*_ are the genotypic values for one homozygote and heterozygote, respectively, at locus i. In turn, the quantity *m*_*d*_ can be directly defined as *m*_*di*_ = *d*_*i*_. The matrices **U** and **S** are defined based on the values 0, 1 and 2 for the number of one of the alleles at the *i*^th^ marker locus in a diploid individual. The correct parameterization of **U** and **S** is as follows, according to the marker genotypes at a locus m.

$$U=\{\begin{array}{c} MM:2-2p\;\to 2q\;\\ Mm:1-2p\;\to q-p\\ mm:0-2p\;\to -2p \end{array}$$

$$S=\{\begin{array}{c} MM:0\;\to -2q^{2}\;\\ Mm:1\;\to 2pq\\ mm:0\to -2p^{2} \end{array}$$

The covariance matrix for the additive effects is given by $G_{a}\sigma_{a}^{2}$ = *Var*(*Um*_*a*_) = $UU^{\prime }\sigma_{m_{a}}^{2}$, which leads to: $G_{a}=UU^{'}/\left(\sigma_{m_{a}}^{2}/\;\sigma_{a}^{2}\right)=UU^{\prime }/\overset{n}{\underset{i=1}{\sum }}\left[2p_{i}\left(1-p_{i}\right)\right]$, as $\sigma_{a}^{2}$ = $\sum_{i=1}^{n}\left[2p_{i}\left(1-p_{i}\right)\right]\sigma_{m_{a}}^{2}$. The covariance matrix for the dominance effects is given by ***G***_***d***_ = $Var\left(Sm_{d}\right)\;SS^{'}\sigma_{m_{d}}^{2}$. Thus, $G_{d}\sigma_{d}^{2}=SS^{\prime }/\left(\sigma_{m_{d}}^{2}/\;\sigma_{d}^{2}\right)=SS^{'}/\sum_{i=1}^{n}\left[2p_{i}\left(1-p_{i}\right)\right]$ as $\sigma_{d}^{2}\;=\sum_{i=1}^{n}\left[2p_{i}\left(1-p_{i}\right)\right]\sigma_{m_{d}}^{2}$. The additive (i.e., narrow-sense) heritability was calculated as ${\hat{h}}_{\alpha }^{2}={\hat{\sigma }}_{\alpha }^{2}/\left({\hat{\sigma }}_{\alpha }^{2}+{\hat{\sigma }}_{d}^{2}+{\hat{\sigma }}_{e}^{2}\right)$ and the dominant heritability as ${\hat{h}}_{d}^{2}={\hat{\sigma }}_{d}^{2}/\left({\hat{\sigma }}_{\alpha }^{2}+{\hat{\sigma }}_{d}^{2}+{\hat{\sigma }}_{e}^{2}\right)$. The additive-dominance GBLUP method was fitted using GenomicLand software [25] via REML through mixed model equations.

#### Artificial neural network

The ANN is composed by a combination of neurons in a single or multiple layers. A vector of real values enters as input in each neuron, with the values 0, 1 and 2, which are computed the weighted average of these values followed by a transformation, then the output of neurons can be directly fed as input into other neurons in the next layer [26].

One of the most common families of architectures for connecting neurons into a network is the feed-forward, which can have multiple layers [27]. This architecture is composed by an input layer (IL), *j* = 1,2, …, *J* hidden layers (HL), and an output layer (OL). The IL is composed by *n*_*il*_ neurons corresponding to the number of markers, the HL are composed by *n*_1_, *n*_2_, …, *n*_*j*_ neurons respectively, and the OL is composed by *n*_*ol*_ neurons representing the output values of the application. In this architecture every neuron of the layer *j* is connected only to the neurons of the layer j +1 producing matrixes of weights *W*^*i*^, where the output is generated by a linear combination of the last HL.

**Fig 1:**
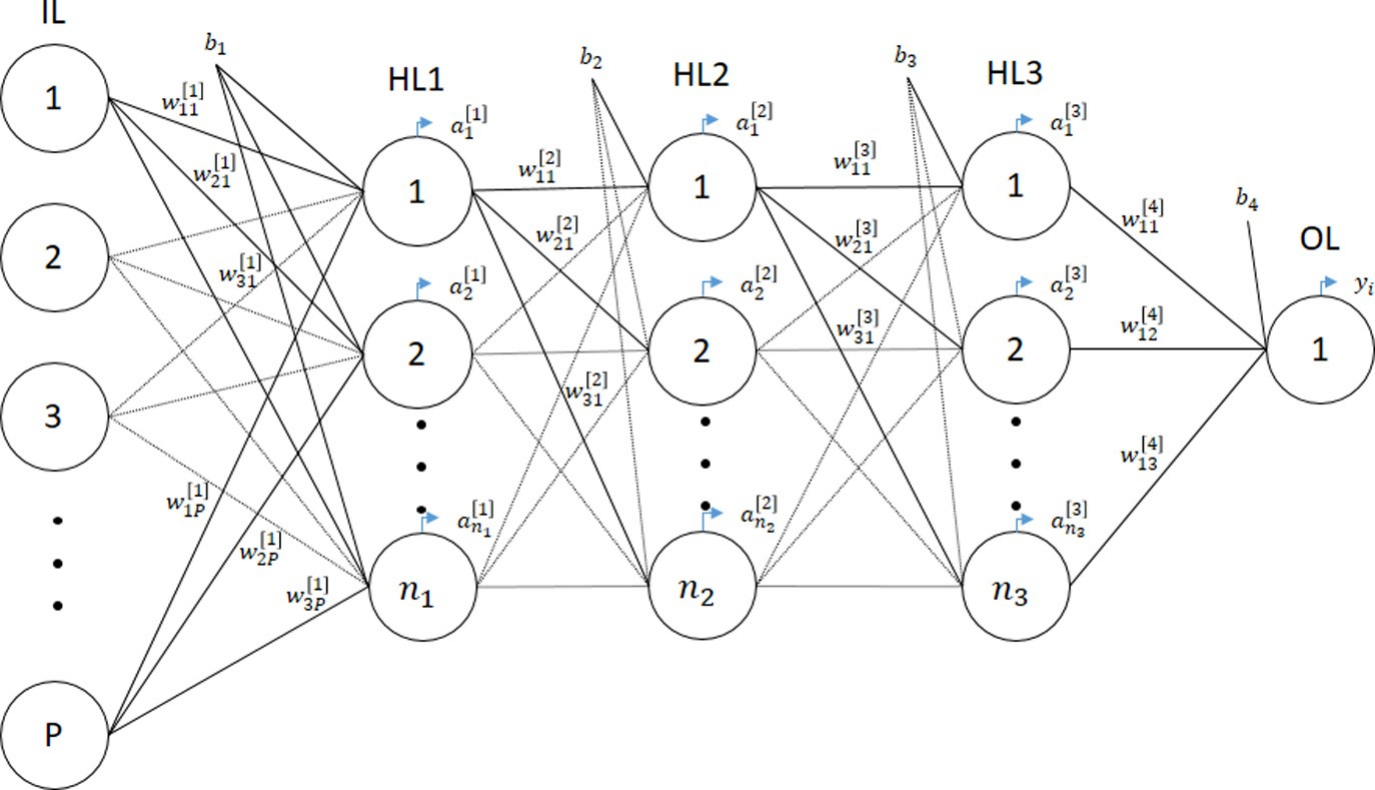
Multilayer perceptron architecture. Feed forward neural network architecture with three hidden layers.

As we can see in Fig 1, the output of the neurons in the first HL (HL1) is given by $a_{i}^{\left[1\right]}=f(\sum_{t=1}^{P}w_{1t}^{\left[1\right]}x_{ti}+b_{1})$, in the second HL (HL2), the outputs of the neurons is given by a linear combination of the outputs from HL1: $a_{i}^{\left[2\right]}=g(\sum_{t=1}^{n_{1}}w_{1t}^{\left[2\right]}a_{t}^{\left[1\right]}+b_{2})$. The third HL (HL3) output is obtained using the same thoughts we use to obtain those from HL2. Finally, the outputs from the OL is obtained by $y_{i}=z(\sum_{t=1}^{n_{3}}w_{1t}^{\left[4\right]}a_{t}^{\left[3\right]}+b_{4})=y_{i}=z(\sum_{t=1}^{n_{3}}w_{1t}^{\left[4\right]}h\left(\sum_{t=1}^{n_{2}}w_{1t}^{\left[3\right]}a_{t}^{\left[2\right]}+b_{3})+b_{4}\right)$ $=z(\sum_{t=1}^{n_{3}}w_{1t}^{\left[4\right]}h\left(\sum_{t=1}^{n_{2}}w_{1t}^{\left[3\right]}g\left(\sum_{t=1}^{n_{1}}w_{1t}^{\left[2\right]}a_{t}^{\left[1\right]}+b_{2})+b_{3}\right)+b_{4}\right)$ $=z(\sum_{t=1}^{n_{3}}w_{1t}^{\left[4\right]}h\left(\sum_{t=1}^{n_{2}}w_{1t}^{\left[3\right]}g\left(\sum_{t=1}^{n_{1}}w_{1t}^{\left[2\right]}f\left(\sum_{t=1}^{P}w_{1t}^{\left[1\right]}x_{ti}+b_{1})+b_{2}\right)+b_{3}\right)+b_{4}\right)$.

Once an ANN demands high computational processing, it is necessary the use of methodologies to reduce the dimensionality of the data [28]. The reduction of the markers was made by Bagging decision tree. This procedure is an ensemble methodology consisting of training many decision trees built using a random part of the same original data. The variables that, on average, reduces more the residual sum of squares (RSS) are classified as the most important variables. We selected the variables that accumulated 70% of the total importance and used them in the ANN. The network structure considers 1,302 markers as input for resistance to coffee leaf and 1,086 markers as inputs for yield, three hidden layers, and the output that predicts traits. The ANNs architecture uses the backpropagation as a learning algorithm [29] and the logistic function as activation function. The three hidden layers varied from 1 to 40 neurons, and the architecture was chosen according to the best predictive ability.

To estimate the heritability and SNP effects, the relative importance (RI) of markers were obtained. Olden et al. [30] proposed a methodology that uses all the connection weights even when the ANN has multiple hidden layers to obtain the RI. To calculate the vector of RI of all markers, the connection weights matrices were multiplied. Considering ***W***^[*i* = 1]^ as the matrix of estimated weights connecting the (*j*—1)^*th*^ layer to the *j*^*th*^ layer where *j* is the number of layers of the ANN, the RI is obtained multiplying ***W***^**[j]**^ * *W*^**[2]**^ * …*W*^**[j-1]**^. To estimate the additive and dominant SNP effect vectors (***β***_***a***_ *and* ***β***_***d***_) using RI, a linear approximation adapted from [31] was used. The estimators are given by $\hat{\beta }=ZM^{\prime }{\left(MZM^{\prime }\right)}^{-1}\hat{y}$ changing only the codification of the matrix ***M*** to obtain the additive or dominant effect, ***Z*** is a diagonal matrix composed by the RI values, the matrix ***M*** is the matrix of markers and $\hat{y}$ is the genomic estimated breeding values (GEBV) from ANN.

To estimate heritabilities, the additive and dominant variance $\left(\sigma_{\alpha }^{2}\;\text{and}\;\sigma_{d}^{2}\right)$ were estimated using ${\hat{\beta }}_{\alpha }$ and ${\hat{\beta }}_{d}$ in the following equations: ${\hat{\sigma }}_{\alpha }^{2}=\overset{P}{\underset{j=1}{\sum }}2p_{j}\left(1-p_{j}\right){\hat{\beta }}_{\alpha_{j}}^{2}$ and ${\hat{\sigma }}_{d}^{2}=\overset{P}{\underset{j=1}{\sum }}{\left(2p_{j}\left(1-p_{j}\right)\right)}^{2}{\hat{\beta }}_{d_{j}}^{2}$. The residual variance $\left(\sigma_{e}^{2}\right)$ was estimated through the difference of the real phenotype and GEBV, thus ${\hat{\sigma }}_{e}^{2}=Var\left(\hat{e}\right)$, being $\hat{e}=y-\hat{y}$.

## Results

**Fig 2:**
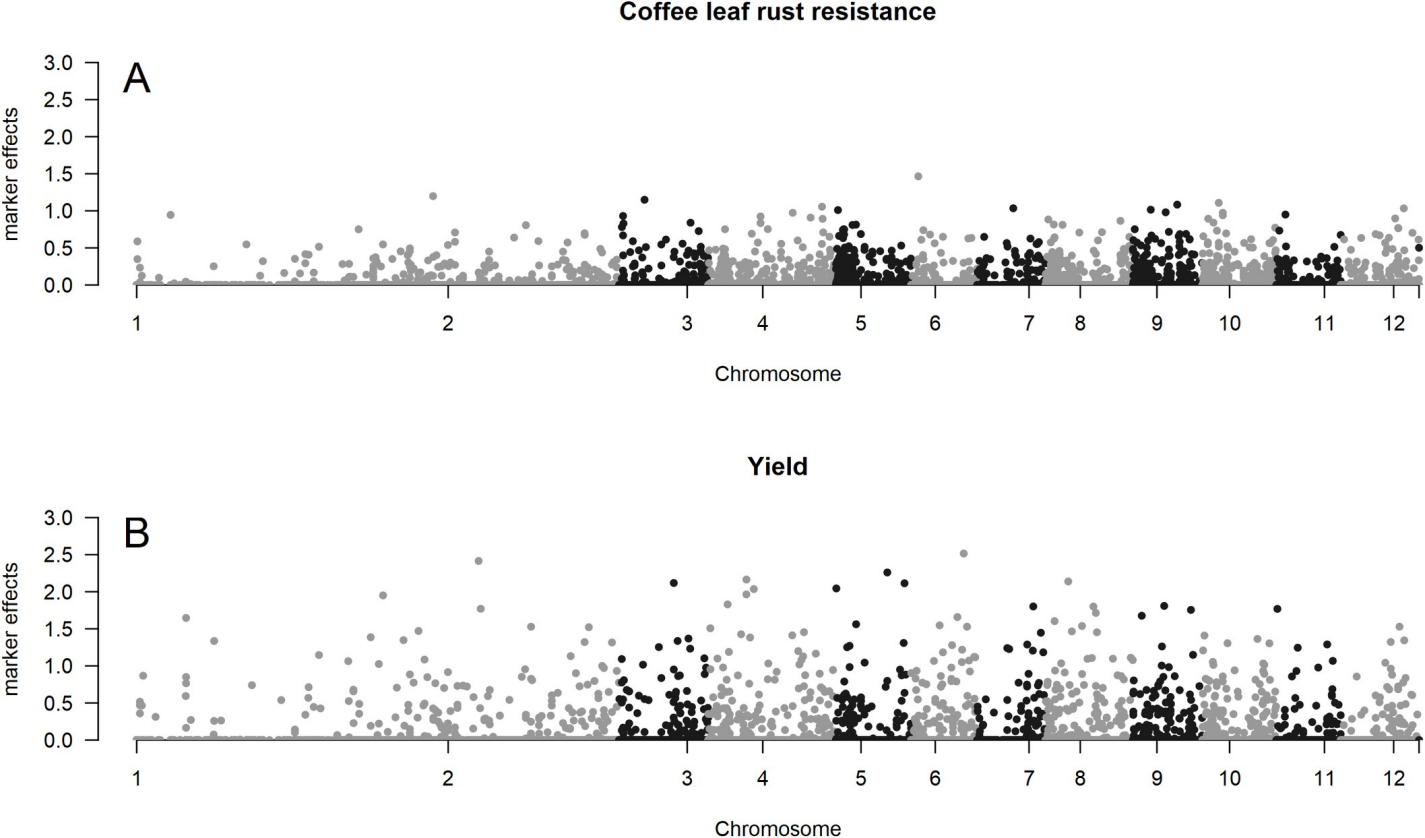
Manhattan plot. A, Manhattan plot showing the effects (in absolute terms) of each marker for coffee leaf rust resistance according to the chromosome position. B, Manhattan plot showing the effects (in absolute terms) of each marker for yield according to the chromosome position.

The input layer (IL) was composed of a genotype matrix **X** with 165 rows (plants) and 1302 columns (markers) for coffee leaf rust resistance. For yield, the matrix was made up of 165 rows and 1086 markers. The markers were selected using bagging. After reducing dimensionality, 64,000 neural networks were performed, with each hidden layer ranging from 1 to 40 neurons, and the ANN was chosen based on the best predictive ability. For yield, the best ANN has 4, 15, and 33 neurons for the first, second, and third hidden layers, respectively. For coffee leaf rust resistance, the best ANN has 13, 20, and 24 neurons for the first, second, and third hidden layers, respectively. In Fig 2, we can observe the map of each trait with the effects (in absolute terms) of each marker estimated by the ANNs cited above.

**Fig 3:**
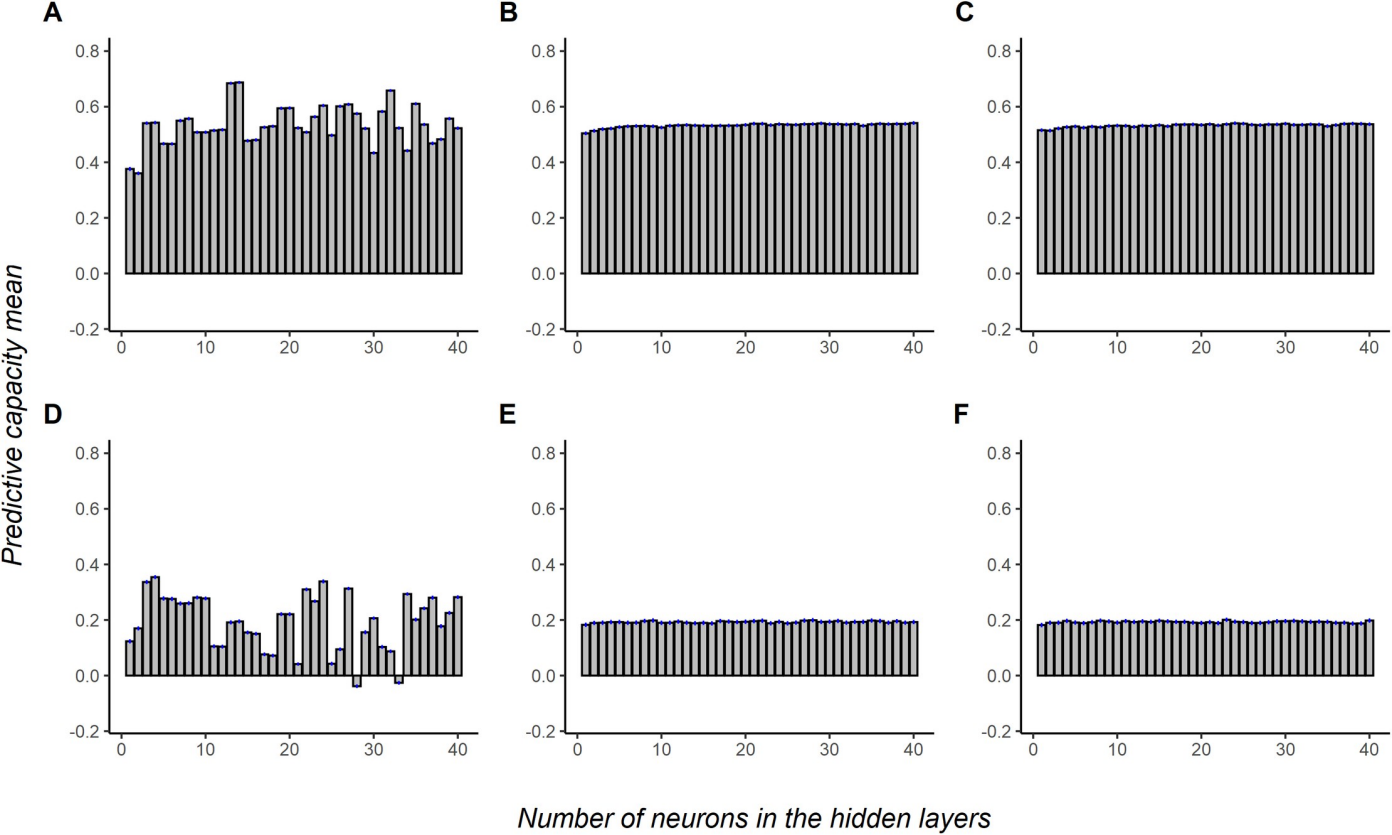
Average predictive capacity of the neural networks according to the numbers of neurons in each hidden layers. A, B, and C are the average predictive capacity when varying the number of neurons in the first, second, and third hidden layers, respectively, for coffee leaf rust resistance in coffee Canephora. D, E, and F are the predictive capacity overage when varying the number of neurons in the first, second, and third hidden layers, respectively, for yield in coffee Canephora.

The predictive ability mean was calculated (Fig 3) by fixing the number of neurons in one HL and varying the number of neurons in the other. The data showed that in Fig 3, the predictive ability is more affected when we change the number of neurons in the first hidden layer. In the second and the third hidden layers, the average predictive ability does not change significantly as we change the number of neurons.

**Fig 4:**
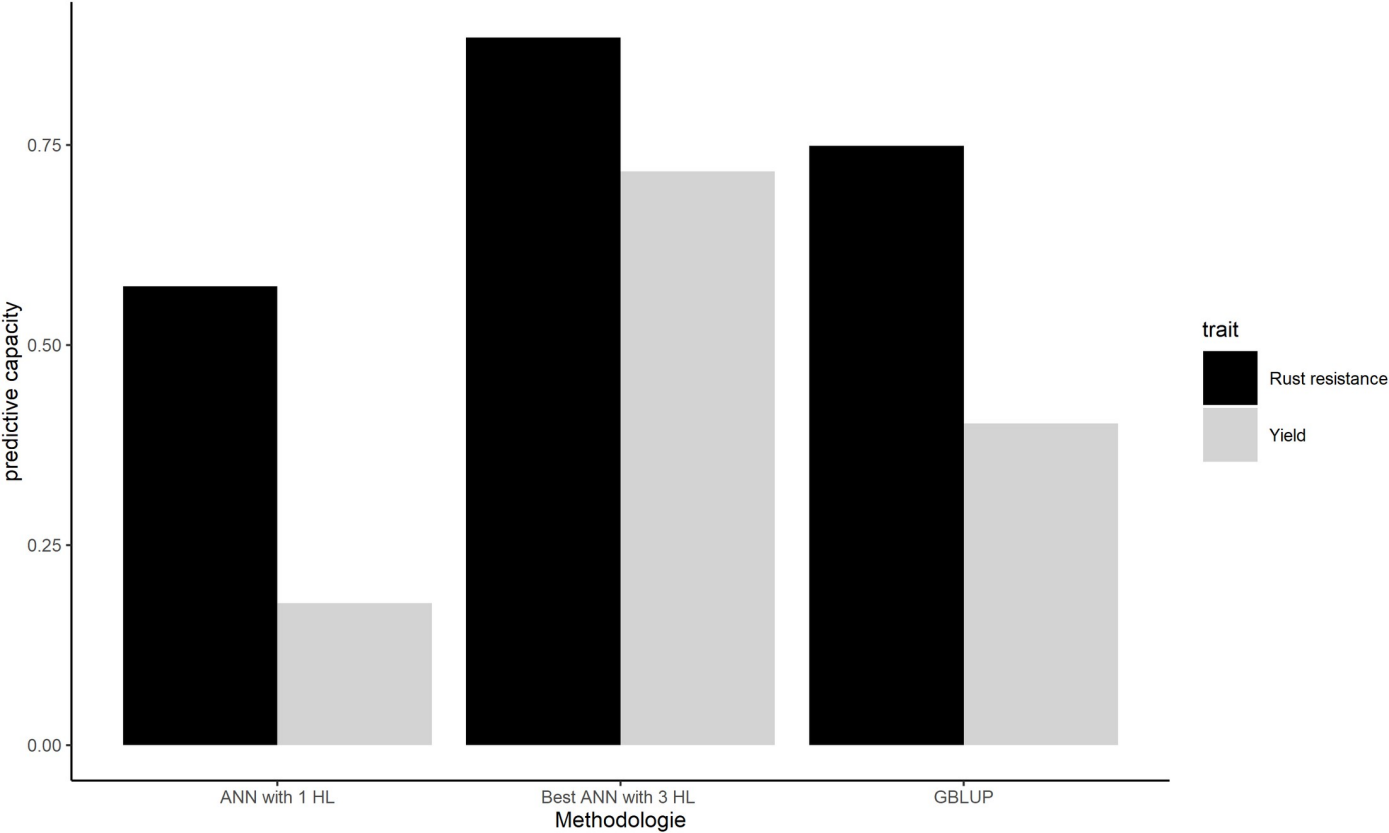
Estimated predictive ability. Yield’s estimated predictive ability and coffee leaf rust resistance’s estimated predictive ability according to artificial neural network with 1 and 3 hidden layers and Genomic BLUP (GBLUP).

The chosen ANNs were compared with GBLUP and with the simplest ANN containing one hidden layer with one neuron and the logistic function as activation function according to predictive ability. The most complex ANNs showed a better predictive ability, 0.72 and 0.88 for yield and coffee leaf rust resistance, respectively, indicating that the traits are complex. The ANNs with a single HL with one neuron showed the worse predictive ability, 0.18 and 0.57 for yield and coffee leaf rust resistance, respectively (Fig 4). The ANNs has the ability to capture non-additive effects as dominance and espistasis [1, 10, 11]. It occurs because the interactions between the markers are implicit in the neuron´s outputs.

For both traits, the additive and dominance heritabilities captured by ANN with 3HL (ANN/3HL) were similar to those obtained by GBLUP (Table 1). The ANN with 1HL (ANN/1HL) showed only additive heritability from coffee leaf rust resistance was similar to the other methodologies.

**Table 1:** Estimates of additive and dominance heritabilities.

	Yield			Rust resistance		
	ANN/1HL	ANN/3HL	GBLUP	ANN/1HL	ANN/3HL	GBLUP
$h_{a}^{2}$	0.07	0.25	0.26	0.55	0.67	0.55
$h_{d}^{2}$	0.02	0.06	0.05	0.45	0.30	0.22

ANN/1HL, an artificial neural network with one hidden layers; ANN/3HL, an artificial neural network with three hidden layer; GBLUP, genomic best linear unbiased predictor; $h_{a}^{2}$, additive heritability; $h_{d}^{2}$, dominance heritability.

**Fig 5:**
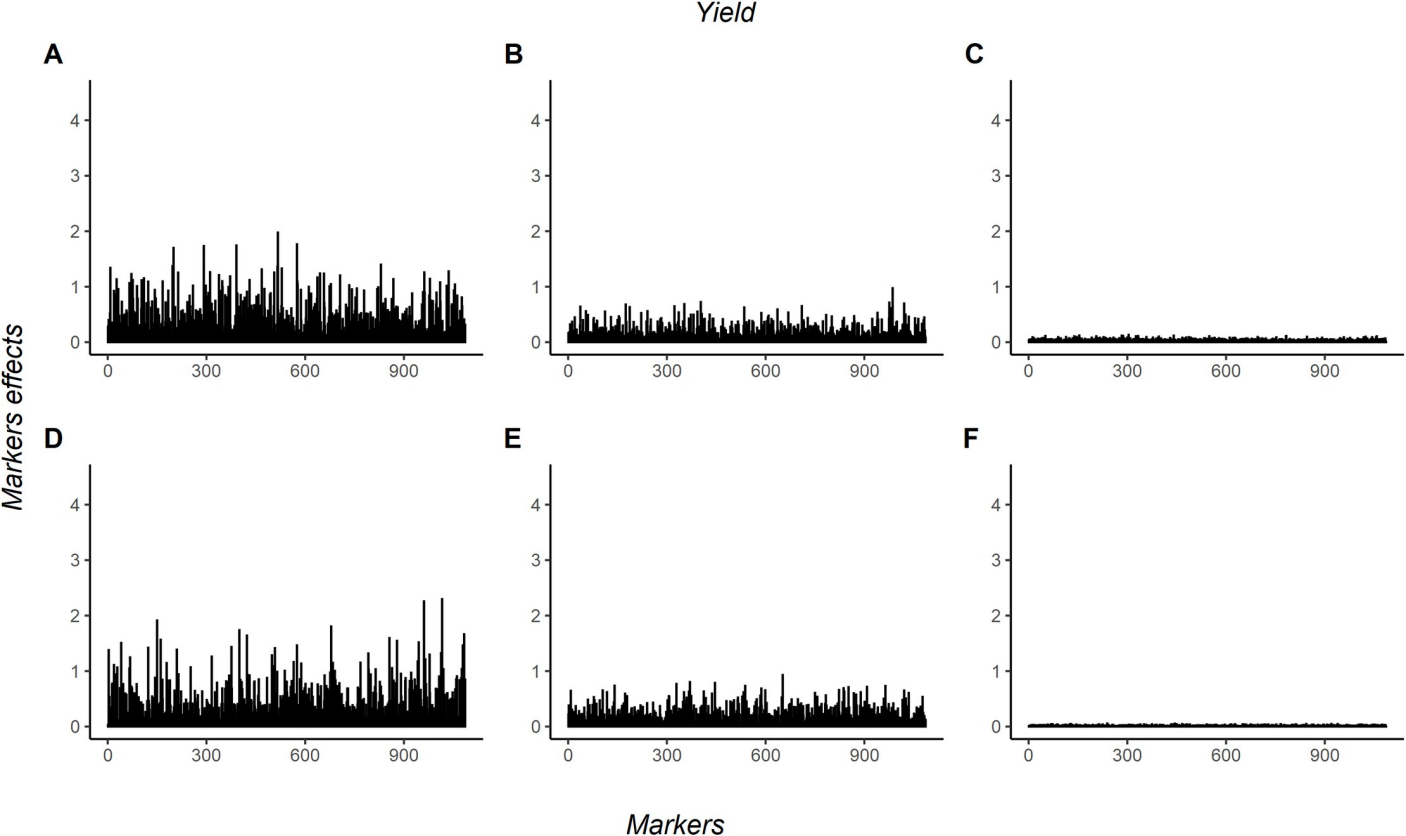
Additive and dominance markers effects for yield in coffee canephora. 1086 markers effects for yield in coffee Canephora. A, B and C are the additive markers effects estimated by a neural network with three hidden layers, a neural network with one hidden layer, and GBLUP, respectively. D, E, and F are the dominance markers effects estimated by a neural network with three hidden layers, a neural network with one hidden layer, and GBLUP, respectively.

The marker effects were estimated using linear approximation [31] based on the method of Olden et al. [30] for ANN. For GBLUP, the marker effects were estimated through a fitted regression model. The absolute values of marker effects from the yield trait are plotted in Fig 5. For this trait, ANN/3HL obtained bigger values than other methodologies evaluated.

**Fig 6:**
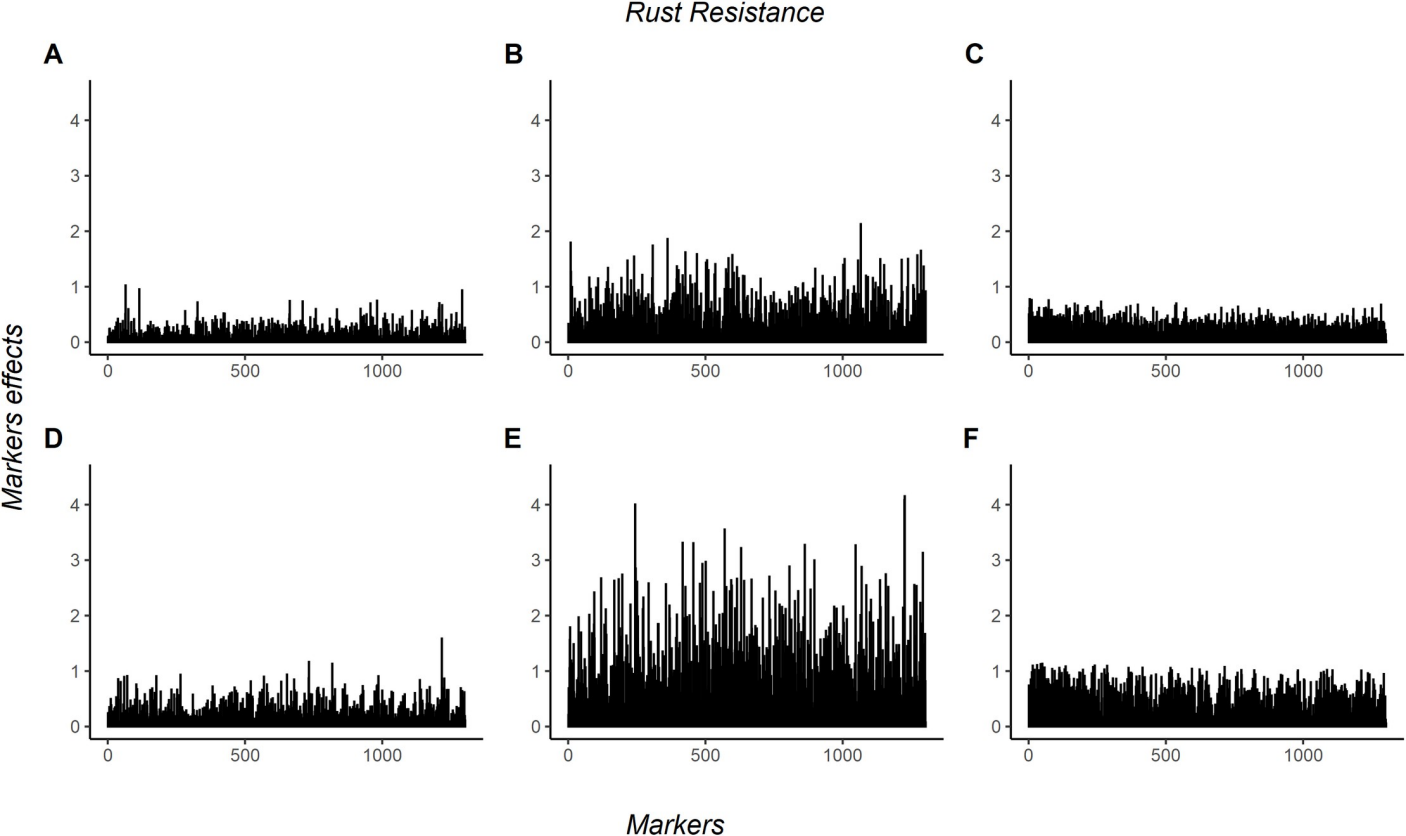
Additive and dominance markers effects for coffee leaf rust resistance in coffee canephora. 1302 markers effects for coffee leaf rust resistance in coffee Canephora. A, B and C are the additive markers effects estimated by neural network with three hidden layers, neural network with one hidden layer, and GBLUP, respectively. D, E, and F are the dominance markers effects estimated by neural network with three hidden layers, neural network with one hidden layer, and GBLUP, respectively.

The absolute values of marker effects from the coffee leaf rust resistance trait are in Fig 6. For this trait, ANN/1HL obtained bigger values than other methodologies evaluated. In both traits, there is not a strong pattern when comparing the important markers among the methodologies.

Looking at the top 10% larger marker effects in each methodology (Table 2), the concordance rate (CR) among additive marker effects was bigger than dominance marker effects. For the yield trait, the CR between ANN/1HL and GBLUP for additive marker effects was bigger (0.14), and between GBLUP and ANN/1HL for dominance, marker effects were the lowest (0.06). For rust resistance, the biggest CR was between ANN/1HL and GBLUP for the additive marker (0.12), the lowest CR was between GBLUP and ANN/1HL for dominance marker effects (0.05).

**Table 2:** Concordance of top 10% bigger marker effect among methodologies, in upper triangular matrix refers to additive marker effects, in lower triangular matrix refers to dominance marker effects.

Methodologies	Yield			Rust Resistance		
	ANN/3HL	ANN/1HL	GBLUP	ANN/3HL	ANN/1HL	GBLUP
ANN/3HL	109	12	13	130	13	15
ANN/1HL	13	109	15	14	130	16
GBLUP	8	6	109	11	7	130

ANN/3HL, Artificial neural network with three hidden layers; ANN/1HL, Artificial neural network with one hidden layer; GBLUP, Genomic Best Linear Unbiased Prediction.

## Discussion

The use of ANN for predicting the individual genetic merit of plants considering yield and coffee leaf rust resistance in *Coffea canephora* was efficient. The ANN/3HL presented higher values of predictive ability compared with those obtained by GBLUP, a result also obtained by Glória et al. [1], Waldmann [32] and Maldonado [7]. Indeed, the better result was expected since the ANN allows to estimate the functional relationships between the variables using nonlinear functions [33]. Thus, the ANN allows great flexibility to handle different types of complex non-additive effects such as dominance and epistasis [34]. The interactions between inputs (SNPs genotypes) and between inputs and the output (phenotypic observations) are naturally modelling from the data. In other words, differently than the traditional methods proposed for genomic selection [11, 35], ANN does not require a priori assumptions about the model relationships allowing to infer the trait architecture directly from the data set [1, 11, 36].

The heritability estimated by ANN/3HL for yield ($h_{a}^{2}$ = 0.25; $h_{d}^{2}$ = 0.06) and coffee leaf rust resistance ($h_{a}^{2}$ = 0.67; $h_{d}^{2}$ = 0.31) were similar to those obtained by GBLUP (yield - $h_{a}^{2}$ = 0.26; $h_{d}^{2}$ = 0.05; coffee leaf rust resistance - $h_{a}^{2}$ = 0.55; $h_{d}^{2}$ = 0.22). In addition, these estimates were consistent with those reported in the literature. The heritability estimate for yield was within the range of estimates for coffee (0.15–0.79 [37]). For coffee leaf rust resistance, the estimate was close to that reported by Alkimin et al. [37] (0.37).

Glória et al [1] considering only additive effects showed that it is possible to obtain estimates from heritabilities through fitting an ANN composes by one layer, one neuron, and identity activation function. However, for some species, for example maize [38, 39], eucalyptus [40, 41], cotton [42, 43], rice [44, 45], pinus [16, 46] and coffee [47, 48], where there is commercial interest in hybrids, the contribution of dominance presents importance. In fact, an ANN composed by one layer, one neuron, and identity activation function can seem like multiple regression. Differently from [1], the ANN/3HL fitted in this work presents more than one hidden layer, and the activation function is not the identity. Nevertheless, the ANN/3HL was able to obtain heritability estimates similar to those obtained by GBLUP. Therefore, besides increasing the predictive ability, the ANN/3HL allows to access the marker effects and consequently the heritability estimate.

A different pattern in marker effects was obtained in the two traits (Figs 5 and 6). A bigger dominance markers effects were observed for yield when compared with the additive marker effect. In comparison, the additive marker effects were bigger than dominance for coffee leaf rust resistance. This can be explained due yield be a polygenic trait and coffee leaf rust resistance oligogenic. According to Cruz [49], when the trait is polygenic, and there is none or fewer dominance, the phenotype distribution becomes symmetric and starts to obtain asymmetry as the dominance starts to increase. Observing the histogram of both traits (S1 Fig), we see that yield has symmetry distribution and coffee leaf rust resistance an asymmetry distribution.

An issue related to using an ANN approach is the computational cost [50]. Once it is necessary to choose the best network topology, the ANN fitting requires a high computational cost. The ANN/3HL was 409.36 and 1331.49 times slower than GBLUP for yield and coffee leaf rust resistance, respectively. Some approaches can be used to minimize the computational cost. For example, it is possible to reduce the number of inputs of an ANN using some reduction dimensionality methods [51]. Other approaches to select markers used in this work are based on machine learning [52]. Sousa et al. [53] used bagging to select the most important markers. However, since, in general, the number of markers is huge in genomic selection problems, the use of a methodology to reduce the computational cost cannot be effective.

## Conclusions

The Artificial Neural Network was able to access the marker effects and heritability estimates from additive-dominance genomic architectures by neural networks in *Coffea canephora*. In addition, considering the estimates of predictive ability, ANN/3HL presented better results compared with those obtained from GBLUP and ANN/1HL.

## Supporting information

S1 Fig Histogram. Histogram of yield and rust resistance.
(TIF)
