# Supplementary figures and images for: Marker effects and heritability estimates using additive-dominance genomic architectures via artificial neural networks in Coffea canephora
Source: PLoS One. 2022 Jan 26;17(1):e0262055. doi: 10.1371/journal.pone.0262055 (PMC8791507; doi:10.1371/journal.pone.0262055)

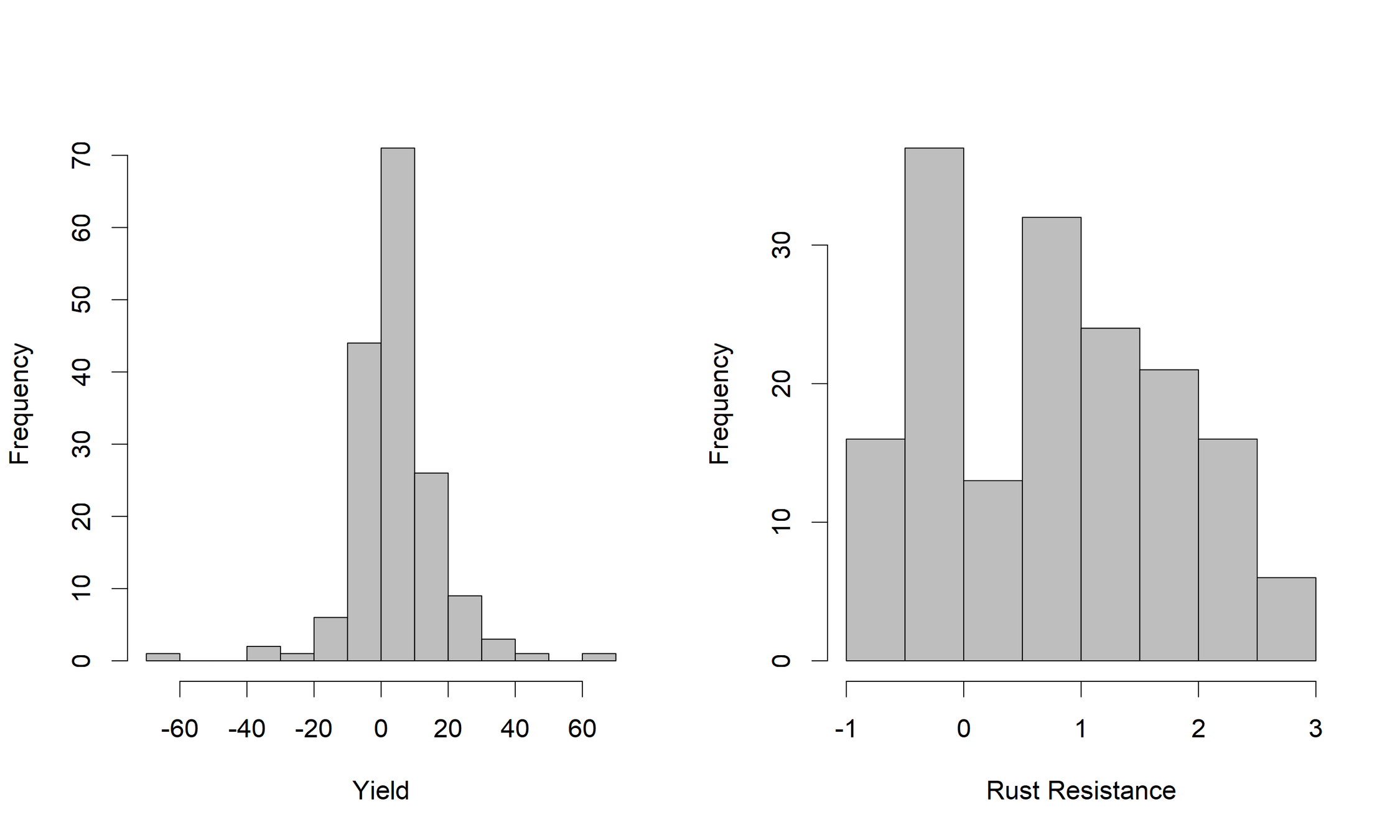

Supplement: S1 Fig — Histogram of yield and rust resistance. (TIF) [file pone.0262055.s001.tif]
